# High affinity of cadmium and copper to head kidney of common carp (*Cyprinus carpio* L.)

**DOI:** 10.1007/s10695-013-9819-1

**Published:** 2013-06-12

**Authors:** Elżbieta Kondera, Katarzyna Ługowska, Piotr Sarnowski

**Affiliations:** Department of Animal Physiology, University of Natural Sciences and Humanities, Prusa 12, 08-110 Siedlce, Poland

**Keywords:** Head kidney, Bioaccumulation, Cadmium, Copper, Fish, Toxicity

## Abstract

The aim of the present study was to evaluate bioaccumulation of metals in various tissues of the freshwater fish *Cyprinus carpio* L. exposed to cadmium and copper (a xenobiotic and a microelement). The fish were subjected to short-term (3 h, Cd-S and Cu-S) or long-term (4 weeks, Cd-L and Cu-L) exposures to 100 % 96hLC_50_ or 10 % 96hLC_50_, respectively. Blood, gill, liver, head and trunk kidney were isolated weekly from 5 fish of each group for 4 weeks (post-short-term exposure and during long-term exposure). Atomic absorption spectrophotometry technique was applied to measure concentrations of metals (Cd and Cu) in fish tissues. Initial concentrations of copper in fish tissues were higher than levels of cadmium. Cadmium and copper levels increased in all tissues of metal-exposed fish. After short-term exposures (at higher concentration) and during long-term exposures (at lower concentration), similar changes in metal concentrations were observed. The values of accumulation factor (ratio of final to initial metal concentration) were higher for cadmium as compared to copper. Comparison of metal levels and accumulation factors in various tissues revealed that cadmium and copper showed very high affinity to head kidney of common carp (higher than to other tissues), but accumulation factors for cadmium in trunk, head kidney and liver were much higher than for copper. The concentrations of copper in organs of Cu-exposed fish increased only slightly and quickly returned to the control level, which shows that fish organism easily buffered metal level. On the other hand, concentrations of cadmium considerably increased and remained elevated for a long time which suggests that activation of mechanisms of sequestration and elimination of cadmium required more time.

## Introduction

In clean natural waters, concentrations of cadmium and copper are very low, but in contaminated waters, they may increase as a result of human activities. Copper-containing compounds are used in aquaculture and agriculture: e.g., pesticides, fungicides, herbicides, bactericides (Murray-Gulde et al. 2002; Carvalho and Fernandes 2006). Main source of Cd pollution is industry: mines and foundries, phosphate fertilizer production and electroplating wastes (Wittman and Hu 2002; Bonda et al. 2007).

Copper and cadmium are metals, which are highly toxic to aquatic animals (Jezierska and Witeska 2001; Mendez-Armenta and Rios 2007). Cadmium is a xenobiotic and does not play any known metabolic role. It is genotoxic, mutagenic, carcinogenic and teratogenic (Walker 2000; Gabbianelli et al. 2003; Cavas et al. 2005). On the other hand, copper is an important essential element involved in various metabolic processes, e.g., neurotransmitter function, iron absorption from the intestine or synthesis of hemoglobin—it plays an important role in production of red blood cells (erythropoiesis), it is also a component of many enzymes (Fedeli et al. 2010). Both copper shortage and excess exert adverse effects on organisms. However, the boundary between necessary and toxic concentration of copper is difficult to determine, because toxic potential of metals depends on many factors: physiochemical properties of aquatic environment, fish health or age- and species-specific sensitivity to intoxication (Bozhkov et al. 2010).

Metals get to fish organism different ways: directly from water by gills and skin or by alimentary tract (with food). Most of metal absorbed by fish organism is transported within the body by blood (Pelgrom et al. 1995; Akahori et al. 1999; Bonda et al. 2007). The largest quantities of cadmium and copper are accumulated in metabolically active tissues (e.g., liver, kidney, alimentary tract, spleen), where thay are bound to metallothioneins—MT (Kito et al. 1982; Roesijadi 1994, Roesijadi et al. 1996; Pelgrom et al. 1995; Castano et al. 1998; Hermesz et al. 2001; Calta and Canpolat 2006; Rose et al. 2006; Panchanathan and Vattapparumbil 2006; Wu et al. 2007; Asagba et al. 2008; Dang et al. 2009; Kovarova et al. 2009). The MT plays an important role in the homeostasis of essential metals such as Cu and Zn and the sequestration of nonessential metals, like Cd and Hg (Coyle et al. 2002). MT containing about 25–35 % cysteine, due to which they have high binding capacity for metals. All SH-groups may bind a metal ion; however, about 50 % of metal-binding sites are always saturated with Zn. One MT molecule can sequester 6–7 cadmium molecules (Hamer 1986). Two MT isoforms corresponding to classes MT-1 and MT-2 were isolated from the kidney and hepatopancreas of the common carp (Kito et al. 1986; Hermesz et al. 2001). Metal accumulation depends on tissue metabolism and other factors such as dose of metal, time of exposure, chemical form of metal or species and age of fish (Bielmyer et al. 2006; Bonda et al. 2007).

Accumulation of cadmium and copper in fish tissues has been extensively studied by many authors (e.g., De Conto Cinier et al. 1997; Kraemer et al. 2005; Calta and Canpolat 2006; Celechovska et al. 2007; Karaytug et al. 2007; Singh et al. 2008; Vinodhini and Narayanan 2008; Rauf et al. 2009), but affinity of metals to head kidney *(pronephros*) and their toxicity to this organ are almost unknown (Garofano and Hirshfield 1982; Ghosh et al. 2007; Som et al. 2009; Kondera and Witeska 2012).

Head kidney plays an important role as main hematopoietic organ in most teleost fishes (Fange 1994; Houston et al. 1996; Fijan 2002a, b; Romano et al. 2002; Moritomo et al. 2004; Rombout et al. 2005; Gangopadhyay and Homechaudhuri 2011). *Pronephros* functions also as a secondary lymphoid organ in which large numbers of antibody producing cells reside. Moreover, production of corticosteroids and catecholamines (hormone participating in stress response) takes place in head kidney (Wendelaar Bonga 1997; Hontela 1998). Therefore, hematopoietic, immune and endocrine functions are combined in *pronephros* (Wendelaar Bonga 1997; Weyts et al. 1999), thus cadmium and copper accumulation in head kidney can produce toxic effects on many important physiological processes in fish. Subletalne concentrations of both metals can change hormone levels (Hontela 1998; Lizardo-Daudt et al. 2007; Ramesh et al. 2007; Dangre et al. 2010), immunological mechanisms (Petanova et al. 2000; Jelovcan et al. 2003; Lafuente et al. 2004) or hematological parameters (Kondera and Witeska 2012; Witeska et al. 2010, 2011).

The aim of the present study was to evaluate the bioaccumulation of metals in the most important tissues participating in uptake, transport, metabolism and excretion of metals (blood, gill, liver and trunk kidney), and the head kidney as a key hematopoietic organ of the fresh water *Cyprinus carpio* L. exposed to cadmium and copper.

## Materials and methods

Six-month-old carp juveniles (*C. carpio* L.) of body mass 21.6 ± 8.3 g were harvested in autumn from the rearing pond of the Inland Fisheries Institute in Żabieniec. At the Department of Animal Physiology, University of Natural Sciences and Humanities in Siedlce the fish were acclimated for a month to the laboratory conditions in the flow-through aerated tank, at the temperature 17–18 °C (dissolved oxygen level 66–87 % of saturation, concentration of NO_2_^−^ 0.02–0.06 mg/dm^3^ and NH_4_^+^ 4.6–7.1 mg/dm^3^). The fish were fed Aller Classic 4 mm pellets (30 % protein, 7 % fat, 43 % carbohydrate, 7 % ash, 5 % fiber) once a day at the rate of 2 % of body mass/day. Then, the fish were transferred to 100 dm^3^ aerated aquaria (10 fish in each) and fed Aller Aqua Classic 4 mm (1 % of stock mass/day, once a day before water renewal). Every day three-fourth of water was renewed without disturbing fish. Prior to the experiment, 96-h survival tests were performed, and 96hLC_50_ values were calculated using the probit method for both metals. Concentrations used in the experiment were based on 96hLC_50_ values to ensure the same toxic power of both metals. The fish were subjected to short-term exposures (3-h exposure to 6.5 mg/dm^3^ cadmium or 0.75 mg/dm^3^ copper—100 % of 96hLC50—groups Cd-S and Cu–S) and long-term exposures (4-week exposure to 0.65 mg/dm^3^ cadmium or 0.075 mg/dm^3^ copper—10 % of 96hLC50—groups Cd-L and Cu-L). Experimental solutions were made using CdCl_2_ × 2½H_2_O and CuSO_4_ × 5H_2_O. Control group was kept in clean tap water (0.3–1 μg/dm^3^ of Cd, 2–33 μg/dm^3^ of Cu, pH 7.5–7.6, hardness 179–198 mg/dm^3^ CaCO_3_).

Blood (1 cm^3^), main hematopoietic organ—head kidney and the most important organs participating in uptake, transport, metabolism and excretion of metals (gill, liver and trunk kidney) were sampled weekly for 1 month from 5 fish from each metal-exposed group (total number of fish = 80), and 10 fish in control group. The separated tissues were weighed (the laboratory weight Radwag the wax 40/160 No. 103440) and dried for 48 h at 70 °C. Dried tissues were manually ground in the mortar, transferred to beakers and dissolved in 2 cm^3^ of 69 % HNO_3_ (the Trace the Pur, Merck). After 24 h, 1 cm^3^ of 33 % H_2_O_2_ (Trace Pur, Merck) was added, and the samples were heated for 1 h to boiling in water baths to complete mineralization of tissues. Then, the concentrations of copper (using AAS flame method) and cadmium (using ETAAS method with electrothermal atomization) were measured in all samples in the atomic absorption spectrophotometer (the AAS-30 the Zeiss) in the Institute of Chemistry of Siedlce University of Natural Sciences and Humanities (Oprządek et al. 2006, 2010). Concentrations of metals in fish tissues were calculated according to calibration curves (0.001–0.1 μg Cd/ml and 0.1–0.25 μg Cu/ml). For copper, 1–2 replicates of each sample were analyzed, while for cadmium 3–5. Accuracy and precision of methods applied were evaluated using prawn certified reference materials (GBW 08572). The results showed good accordance with the certified values (93–103 %). Concentrations of metals in fish tissues were calculated per 1 g of fresh mass of each organ, and the results were given as μg/g wet weight. Cadmium and copper accumulation factors—A (the ratio of final to initial metal concentration) were also calculated.

The obtained results were subjected to statistical analysis using the nonparametric *U* Mann–Whitney test, assuming that differences were significant at *p* ≤ 0.05.

The study obtained agreement of the III Local Ethical Committee at the Warsaw University of Life Sciences (No. 41/2008).

## Results

In tissues of fish from the control group, the level of copper was 3.1–38.7 μg/g, while concentrations of cadmium were much lower: 0.1–3.6 μg/g. Concentrations of both metals in the control group were the highest in head kidney, while the lowest levels were observed in trunk kidney (Cd) and gill (Cu).

**Fig. 1:**
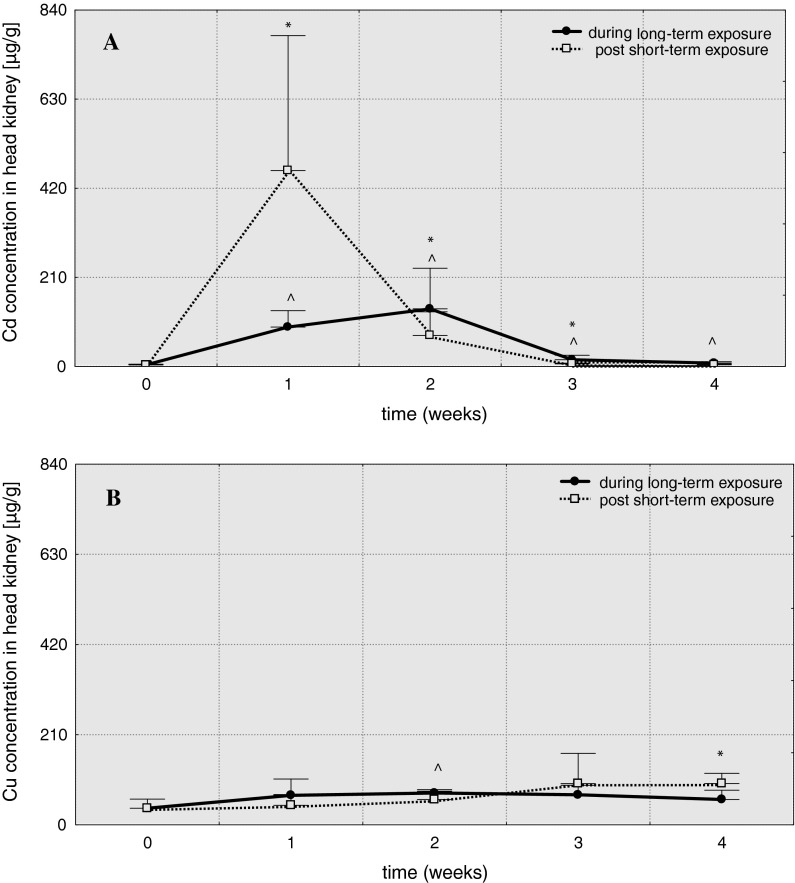
Cadmium (**A**) and copper (**B**) concentrations in head kidney of common carp (μg/g) over 4 weeks post 3-hour exposure to 6.5 mg/dm^3^ of cadmium or 0.75 mg/dm^3^ of copper (96hLC50) and 4-week exposure to 0.65 mg/dm^3^ of cadmium or 0.075 mg/dm^3^ of copper and (10 % 96hLC50), *values significantly different from the control (short-term exposures), **^**values significantly different from the control (long-term exposures), test *U*, *p* ≤ 0.05, *n* = 5

In head kidney, the level of cadmium (Table 1) considerably increased in 1 week after short-term exposure and reached the maximum level: 461.7 μg/g (A = 127.2), then gradually decreased and in 4 weeks the value was similar as in the control (Fig. 1). In fish subjected to long-term Cd-exposure cadmium concentration increased until the 2 weeks (A = 37.4), and then decreased, but remained elevated above the control level until the end of experiment. A significant increase in level of copper (Table 2) was noted only in 4 weeks after short-term exposure (A = 2.5) and in 2 weeks of long-term exposure (A = 1.9).

**Table 1:** Cadmium accumulation factors—A (ratio of final to initial metal concentration) in tissues of common carp over 4 weeks post 3-hour exposure to 6.5 mg/dm^3^ of cadmium (96hLC50)—Cd-S1, Cd-S2, Cd-S3, Cd-S4 and during 4-week exposure to 0.65 mg/dm^3^ of cadmium (10 % 96hLC50)—Cd-L1, Cd-L2, Cd-L3, Cd-L4

Tissues	Experimental groups							
	Cd-S1	Cd-S2	Cd-S3	Cd-S4	Cd-L1	Cd-L2	Cd-L3	Cd-L4
Head kidney	127.2	20.2	1.7	1.1	25.6	37.4	4.4	2.2
Liver	22.8	13.4	2.3	1.3	33.1	14.7	2.3	1.5
Trunk kidney	78.5	129.8	25.1	52.8	73.1	91.5	8.9	40.4
Gill	8.7	6.8	2.2	1.2	5.8	4.6	1.5	1.1
Blood	19.4	17.9	4.4	1.1	18.6	12.6	2.5	1.5

**Table 2:** Copper accumulation factors—A (ratio of final to initial metal concentration) in tissues of common carp over 4 weeks post 3-hour exposure to 0.75 mg/dm^3^ of copper (96hLC50)—Cu-S1, Cu-S2, Cu-S3, Cu-S4 and 4-week exposure to 0.075 mg/dm^3^ of copper (10 % 96hLC50)—Cu-L1, Cu-L2, Cu-L3, Cu-L4

Tissues	Experimental groups							
	Cu-S1	Cu-S2	Cu-S3	Cu-S4	Cu-L1	Cu-L2	Cu-L3	Cu-L4
Head kidney	1.2	1.5	2.5	2.5	1.8	1.9	1.8	1.5
Liver	0.6	0.9	1.8	1.3	1.0	1.4	1.3	1.3
Trunk kidney	0.8	1.0	1.1	1.2	0.9	1.0	1.3	1.4
Gill	0.8	0.9	1.2	1.1	2.3	2.3	2.4	3.2
Blood	0.8	0.9	0.9	0.6	0.8	1.1	1.1	1.1

**Fig. 2:**
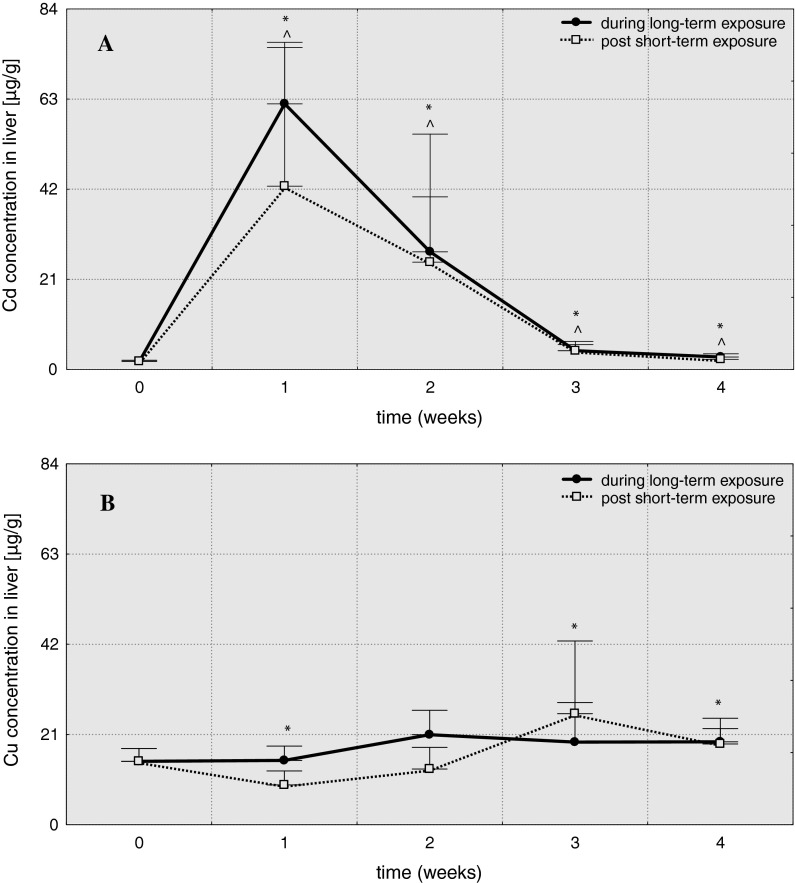
Cadmium (**A**) and copper (**B**) concentrations in liver of common carp (μg/g) over 4 weeks post 3-hour exposure to 6.5 mg/dm^3^ of cadmium or 0.75 mg/dm^3^ of copper (96hLC50) and 4-week exposure to 0.65 mg/dm^3^ of cadmium or 0.075 mg/dm^3^ of copper (10 % 96hLC50), *values significantly different from the control (short-term exposures), **^**values significantly different from the control (long-term exposures), test *U*, *p* ≤ 0.05, *n* = 5

Both short- and long-term exposures of fish in cadmium-contaminated water (Table 1) caused a strong increase in the level of this metal in liver in 1 week of the experiment: Cd-S1 (A = 22.8) and Cd-L1 (A = 33.1) group. Despite a downward tendency, elevated levels of Cd were observed until the end of the experiment (Fig. 2). The increase in copper concentration took place in 2 last weeks of short-term exposure (A = 1.8 in Cu-S3 and A = 1.3 in Cu-S4)—Table 2.

**Fig. 3:**
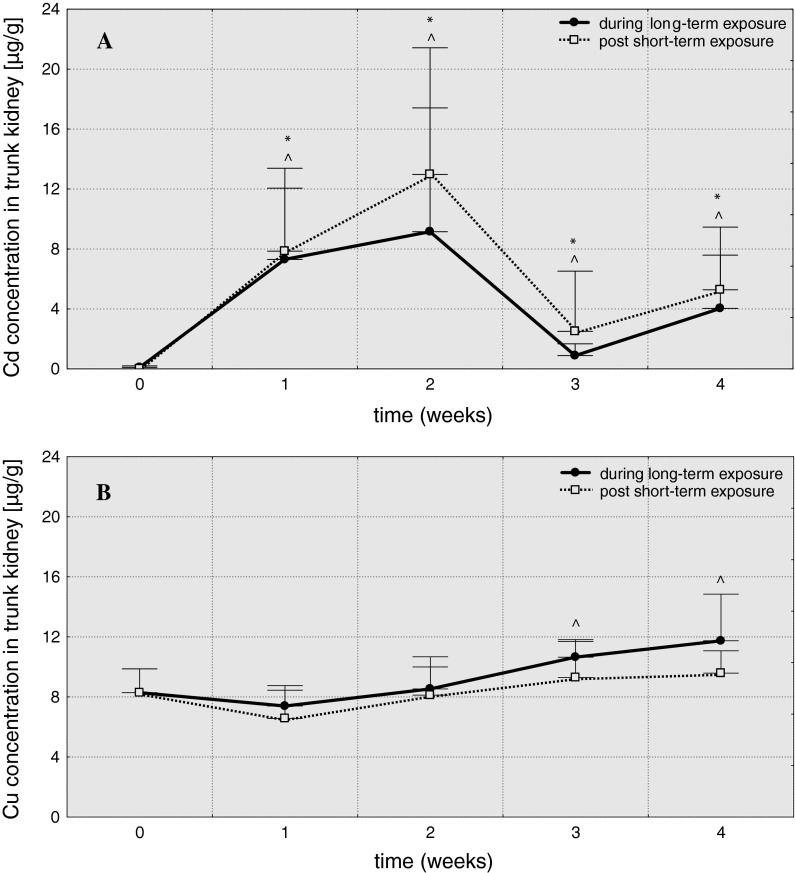
Cadmium (**A**) and copper (**B**) concentrations in trunk kidney of common carp (μg/g) over 4 weeks post 3-hour exposure to 6.5 mg/dm^3^ of cadmium or 0.75 mg/dm^3^ of copper (96hLC50) and 4-week exposure to 0.65 mg/dm^3^ of cadmium or 0.075 mg/dm^3^ of copper (10 % 96hLC50), *values significantly different from the control (short-term exposures), **^**values significantly different from the control (long-term exposures), test *U*, *p* ≤ 0.05, *n* = 5

In trunk kidney (Fig. 3) cadmium concentration increased till 2 weeks when it reached a maximum in Cd-S2 (A = 129.8) and Cd-L2 (A = 91.5). In 3 weeks, a decrease was noted, though Cd concentration was still considerably higher than in the control group (2.5 μg/g in Cd-S3 and 0.9 μg/g in Cd-L3). In the last week of the experiment, concentrations of cadmium in trunk kidney increased again (Cd-S4—5.3 μg/g, Cd-L4—4.0 μg/g). In case of copper, only the long-term exposure caused significant increase in metal level in trunk kidneys in 3 and 4 weeks (to 11.7 μg/g in Cu-L4).

**Fig. 4:**
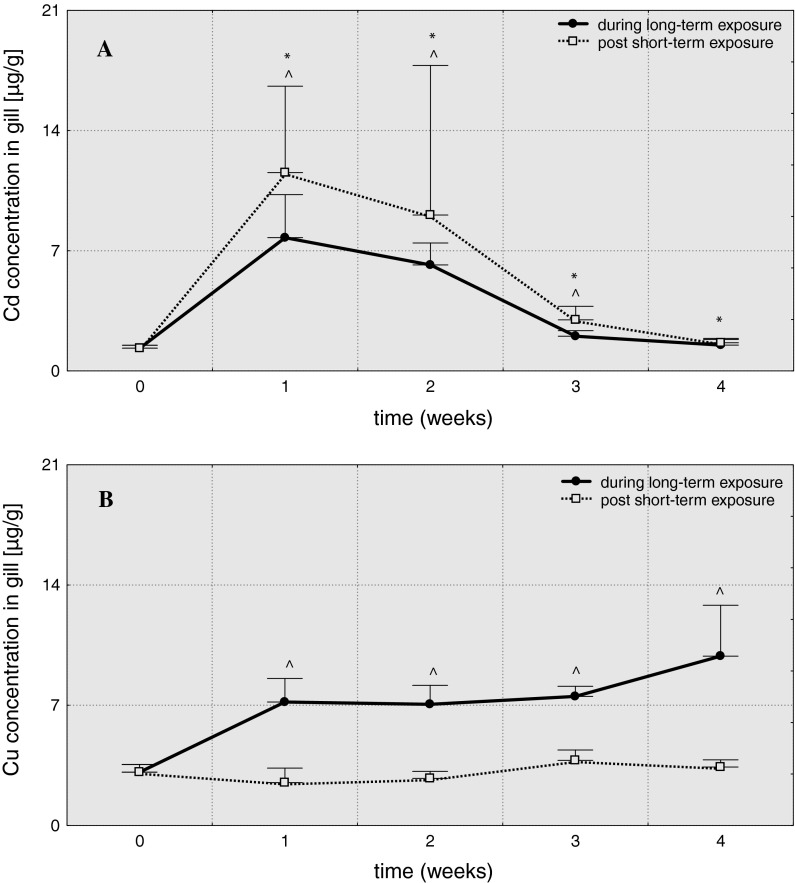
Cadmium (**A**) and copper (**B**) concentrations in gill of common carp (μg/g) over 4 weeks post 3-hour exposure to 6.5 mg/dm^3^ of cadmium or 0.75 mg/dm^3^ of copper (96hLC50) and 4-week exposure to 0.65 mg/dm^3^ of cadmium or 0.075 mg/dm^3^ of copper (10 % 96hLC50), *values significantly different from the control (short-term exposures), **^**values significantly different from the control (long-term exposures), test *U*, *p* ≤ 0.05, *n* = 5

The maximum cadmium levels in fish gills were observed in the first week of the experiment (in Cd-S1 and Cd-L1, respectively: A = 8.7 and A = 5.8)—Table 1.  Then cadmium content in gills decreased, but only in Cd-L4 group returned to the control level, while in Cd-S4 remained elevated (Fig. 4). Significant increase in Cu level was noted in fish subjected to long-term exposure to this metal (A= 2.3–3.2)—Table 2.

**Fig. 5:**
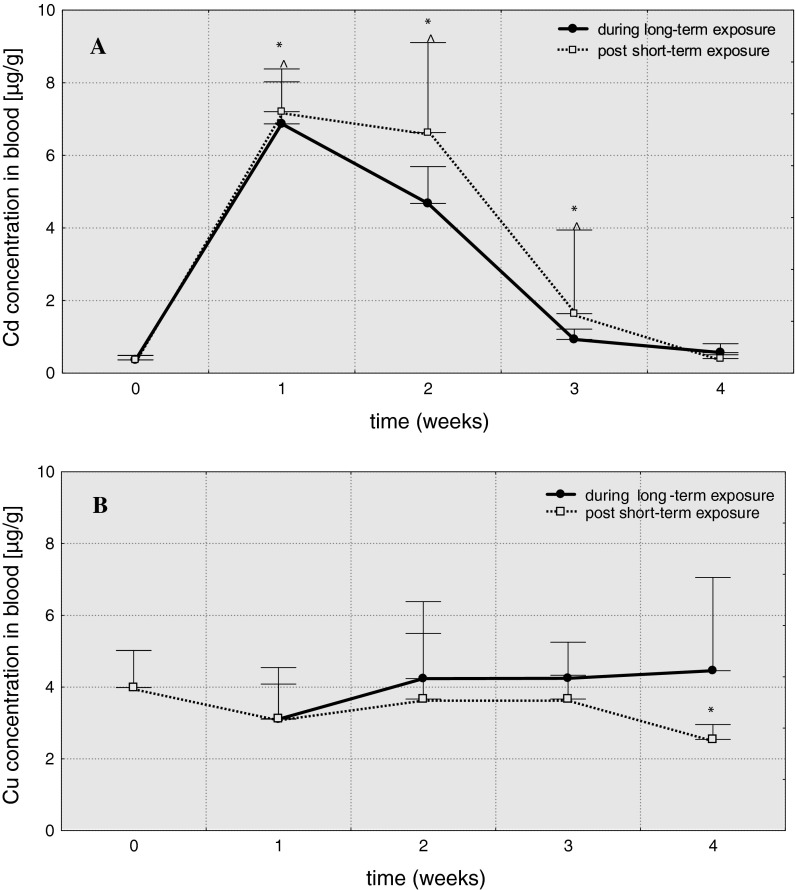
Cadmium (**A**) and copper (**B**) concentrations in blood of common carp (μg/g) over 4 weeks post 3-hour exposure to 6.5 mg/dm^3^ of cadmium or 0.75 mg/dm^3^ of copper (96hLC50) and 4-week exposure to 0.65 mg/dm^3^ of cadmium or 0.075 mg/dm^3^ of copper (10 % 96hLC50), *values significantly different from the control (short-term exposures), **^**values significantly different from the control (long-term exposures), test *U*, *p* ≤ 0.05, *n* = 5

Short-term as well as long-term cadmium exposures caused significant increase in concentrations of this metal in peripheral blood in 1 week (A = 19.4 in Cd-S1, A = 18.6 in Cd-L1)—Table 1. In the subsequent weeks, Cd concentrations gradually decreased and returned to initial level in 4 weeks (Cd-S4, Cd-L4). Blood of fish subjected to copper concentrations of this metal did not significantly increase (A= 0.8–1.1), and even a significant drop of Cu concentration (to 2.5 μg/g) was observed in Cd-S4 group (Fig. 5).

## Discussion

The obtained results show that initial concentrations of copper in fish tissues were higher than the levels of cadmium. According to various authors, cadmium concentration in freshwater fish tissues (intestine, muscles, kidney, gill, skin, spleen, brain, liver) usually does not exceed 2 μg/g (Kraal et al. 1995; Hollis et al. 1999; Panchanathan and Vattapparumbil 2006; Wu et al. 2007; Singh et al. 2008; Tao et al. 2008; Vinodhini and Narayanan 2008; Dang et al. 2009; Isani et al. 2009). According to Calta and Canpolat (2006), natural level of copper in the tissues of cyprinid fishes (*C. carpio*, *Acanthobrama* *marmit*, *Chondrostoma regium*) were in muscles below 5 μg/g, in gill 5–10 μg/g, in skin near 10 μg/g, in gonads 5–20 μg/g, and in liver 15–30 μg/g. Higher natural level of copper in fish in comparison with cadmium obviously results from the fact that Cu is a microelement and thus—natural component of body involved in metabolic processes, while cadmium is a xenobiotic (Ghedira et al. 2010).

Both, after short-term and long-term exposures in water contaminated with cadmium and copper, the levels of metals in carp tissues were considerably higher than their concentration in water which indicates bioaccumulation of these elements in fish organism. Also Radhakrishnan (2010) observed that levels of metals (Fe, Cu, Zn, Mn, Cr) in *Heteropneustes fossilis* gill, liver and muscle were higher than in water. Olowoyo et al. (2011) reported that concentrations of Zn, Fe, Mn, Cr, Ni, Cu, Pb in *C. carpio* and *Clarias gariepinus* liver, gill, muscle, bone were higher compared to the levels of these metals in water and concluded that it was a result of bioaccumulation.

In the present work, both metals showed very high affinity to carp head kidney. Almost no data concerning concentrations of metals in this organ are available in the literature. Garofano and Hirshfield (1982) observed accumulation of cadmium in spleen and head kidney of *Ictalurus nebulosus*. However, Woodling et al. (2001) observed that cadmium and copper were accumulated mainly in fish trunk kidney, while their concentrations in head kidney were considerably lower.

High affinity to head kidney can be source of metal-induced alterations in fish hematopoietic system. Garofano and Hirshfield (1982) reported destruction or elimination of all hematopoietic elements (except for mature erythrocytes) in *pronephros* of cadmium-intoxicated *Ictalurus nebulosus*. Also Saxena et al. (1992) mentioned damage of *Heteropneustis fossilis* hematopoietic tissue caused by this metal. Cadmium was found to downregulate Hb and Epo expression in *Cyprinodon variegatus* larvae under hypoxic conditions (Dangre et al. 2010) which indicates inhibitory effect on erythropoiesis. Changes in hematopoietic activity were also observed in Cu-exposed *Labeo rohita* (Som et al. 2009). Sublethal exposure induced an increase in frequency of erythropoietic and leukopoietic precursor cells, while at lethal conditions, a decrease occurred at the beginning of exposure and was followed by an increase. Som et al. (2009) also reported an increase in frequency of blast cells in copper-exposed *Labeo rohita*, and an increase in apoptotic rate of hematopoietic precursors, while proliferation rate was elevated under sublethal conditions and reduced in fish subjected to lethal exposure. Cadmium and copper can also induce endocrine disruption in head kidney. According to Lizardo-Daudt et al. (2007), a significant drop of cortisol concentration was observed in *Oncorhynchus mykiss* head kidney exposed in vitro to CdCl_2_. Also Hontela (1998) noted a decrease in plasma cortisol level of *Perca flavescens* exposed to cadmium. Significant increase in plasma cortisol and prolactin level of *C. carpio* subjected to acute and sublethal copper treatments was reported by Ramesh et al. (2007).

This study revealed also that copper content in tissues of fish exposed to this metal increased less than cadmium (taking into consideration the difference between concentrations of both metals in water), which indicates that the organism efficiently buffered the level of copper. On the contrary, concentration of cadmium considerably increased, in most cases reaching the maximum in 1 week of tests (similarly, after short-term exposure and during long-term exposure). In subsequent weeks, concentrations of cadmium decreased, but in head kidney, trunk kidney and liver, they remained elevated compared to the control until the end of the experiment. This suggests that activation of mechanisms of sequestration and elimination of cadmium required some time. The maximum concentrations and accumulation factors of cadmium were higher in comparison with copper particularly in head kidney, liver and blood, while in gill and trunk kidney, the levels of both metals were similar.

Al-Nagaawy (2008) reported that accumulation of essential metals (Cu) in *Oreochromis niloticus* gills and muscles was lesser in comparison with xenobiotics (Pb). Considerably higher cadmium concentration compared to copper in gills of *Odontesthes bonariensis* after 16-day exposures was observed by Carriquiriborde and Ronco (2008). Romeo et al. (2000) obtained similar results: After 48 h from injections of cadmium and copper solutions, they noted that the level of cadmium in tissues of *Dicentrarchus labrax* was considerably higher in comparison with the control, while concentration of copper remained unchanged. According to these authors, copper activates detoxification mechanisms of organism more efficiently than cadmium. Kraemer et al. (2005) reported higher Cd accumulation in comparison with Cu as a result of less effective elimination of this element from organism. According to Kalay and Canli (2000), lower accumulation of microelements (Cu, Zn) in relation to xenobiotics (Cd, Pb) can result from better control mechanisms of level and shorter time of half-life in organism and from lower affinity of natural components to tissues.

After short exposures, the levels of metals in some tissues still increased in several weeks after transfer of fish to clean water: The maximum Cu concentration in head kidney and liver was has noted in group Cu-S3 and in trunk kidney in Cu-S4, while the highest level of Cd in trunk kidney was in group Cu-S2. It shows that metals taken up from water were not immediately excreted but were translocated inside the organism. Various dynamics of changes of cadmium and copper levels in the tissues probably resulted from different ways of transportation and accumulation, and pathways of metabolism of each metal.

According to Kraemer et al. (2005), copper and cadmium first show affinity to gill which is main uptake site of waterborne elements, then they are transported via blood to liver and kidney. Metal ions usually accumulated less in gills since they are a temporary target organ of accumulation, and then Cd is transferred to other organs (Wu et al. 2007). Various authors (e.g., Kraal et al. 1995; Jacobson and Reimschuessel 1998; McGeer et al. 2000; Celechovska et al. 2007; Ghedira et al. 2010; Radhakrishnan 2010; Shao et al. 2010) showed that the highest concentrations of copper were noted in fish liver. Liver being main detoxification site in organism is also an organ that bioaccumulates toxic substances, and thus, it usually shows higher concentrations of metals than another tissues (Allen 1995; Olowoyo et al. 2011). Couture and Kumar (2003) suggested that copper and cadmium concentration led to an up-regulation of liver protein metabolism, presumably at least in part for the purpose of metals detoxification. In *Oncorhynchus mykiss*, active regulation of internal Cu levels in response to sublethal waterborne copper, specifically an up-regulation of hepatic turnover and enhanced elimination of Cu via the bile, has been demonstrated by Grosell et al. (1997, 1998). Cadmium is known to disrupt hepatic carbohydrate metabolism, which leads to a decrease in glycogen storage and increased plasma glucose (Soengas et al. 1996).

Cadmium accumulates in tissues of fish easily, showing high affinity to liver and kidneys (e.g., Bentley 1991; Kock et al. 1996; Schultz et al. 1996; Dallinger et al. 1997; De Smet and Blust 2001; Thophon et al. 2003; Reynders et al. 2006; Shalaby 2007; Wu et al. 2007; Ghedira et al. 2010; Cao et al. 2012). De Conto Cinier et al. (1997) observed that Cd reached high concentration in liver earlier than in kidney in carps exposed to this metal. Similar results were obtained in the present study, both after short-term and during long-term exposure. Bonda et al. (2007) reported that after single short exposure to high concentration of cadmium metal was accumulated mainly in liver, but long-term exposures to low concentration of this metal-induced disfunction of kidneys caused by increased accumulation of element in this organ.

A lot of pollutants dissolved in water (also metals) enter to fish organism mainly by gills (Evans et al. 1987; Leguen et al. 2007), across ion channels of respiratory epithelium or protein complex of the chloride cells in gills (Thophon et al. 2003; Galvez et al. 2007). Cadmium uptake involves competition with Ca, Fe and Zn and takes place via their transport systems. According to Verbost et al. (1989), the pivotal mechanism in the cytotoxic action of Cd^2+^ is the inhibition of Ca^2+^ extrusion and disturbance of intracellular Ca^2+^ homeostasis which results in an increase in cytosolic Ca^2+^ to toxic levels. Copper is known to cause osmotic imbalance by reduction of Na^+^/K^+^ ATPase activity (Pelgrom et al. 1995).

Large fraction of copper absorbed by fish organism is transported within the body by blood plasma bound to albumin, histidine, threonine and glutathione (Bettger et al. 1987; Pelgrom et al. 1995) and then is deposited in liver. This gland plays essential role in copper metabolism. In liver, copper is attached to ceruloplasmin. Also metallothioneins (MT) play important role in binding this metal in vertebrates. They protect against toxic action of metals by reducing the concentration of free metal ions to physiological values in the tissues (Roesijadi et al. 1996). MT-complexes play primary function in homeostasis of copper in organism. Due to MT binding Cu accumulated in fish can be eliminated from blood circulation across liver quickly and efficiently. Carbonell and Trazona (1992) suggest that long-term exposures to cooper sulfate do not increase Cu concentrations in fish tissues but modify the relationship between cooper and other essential metals such as iron or zinc. When excess copper penetrate into cells, they are able to displace zinc from thioneins (normally present in cytosol). Copper may also bind to the sulfhydryl groups of several enzymes (e.g., glucose-6-phosphatase, glutathione reductase) thus interfering with their protection of cells from free radical damage. Depending on the level and organism’s demand, copper can be stored, distributed to various tissues or eliminated (Pelgrom et al. 1995).

After absorption, ions of toxic cadmium in circulating blood are mainly absorbed by erythrocytes (they bind to proteins of cells membrane or to hemoglobin). Only a small quantity of Cd in blood is transported bound to albumin, cysteine or glutathione (Bonda et al. 2007). Upon entry into blood plasma, it is distributed throughout the body, with the greatest burdens in the liver and kidneys. At this time, organism activates mechanisms of detoxification (Jonsson and Part 1998). In the liver, Cd not bound to MT induces synthesis of new MT (Pelgrom et al. 1995; De Conto Cinier et al. 1997; Rose et al. 2006; Huang et al. 2007; Wu et al. 2007, Dang et al. 2009; Kovarova et al. 2009; Bozhkov et al. 2010). Riggio et al. (2003) reported that after exposure of *Danio rerio* to cadmium the MT content increased around 30-fold. Wangsongsak et al. (2007) showed that hepatic expression of MT-mRNA increased significantly after Cd exposure of *Pontius gonionotusto*. This operation (synthesis of MT) reduces toxic influence of cadmium on organism and makes possible excretion. According to Roesijadi (1994) and Asagba et al. (2008), cadmium binds with MT (displacing zinc and copper) and easily forms CdMT complexes. Following release from the liver, CdMT reenters the blood stream, is filtered and reabsorbed by the renal proximal tubules. CdMT is transported into lysosomes where the complex is catabolized. This releases cadmium from the complex and free cadmium induces MT synthesis in the kidney (Klaassen and Liu 1997; Brzóska et al. 2003). Then liberated Cd ions are eliminated in the urine. The ability of tissues to synthesize MT protects organism against direct toxicity of metal; however, it causes accumulation of cadmium in organs, which results in its long half-life in organism (Bonda et al. 2007).

In conclusion, comparison of metal levels in various tissues revealed that cadmium and copper showed very high affinity to head kidney of common carp. In organs of fish exposed to copper, the content of this metal increased slightly and quickly returned to the control level, which shows that fish organism easily buffered metal level. However, concentrations of cadmium considerably increased and remained elevated for a long time which suggests that activation of mechanisms of sequestration and elimination of cadmium required more time.
